# Hybrid Clinician-Managers: Exploration of opportunities and challenges in a hospital setting

**DOI:** 10.4102/jcmsa.v3i1.182

**Published:** 2025-08-11

**Authors:** Anita N. Parbhoo, Vera Scott

**Affiliations:** 1 School of Public Health, Faculty of Community and Health Sciences, University of the Western Cape, Cape Town, South Africa; 2 Red Cross War Memorial Children’s Hospital, Western Cape Department of Health and Wellness, Cape Town, South Africa

**Keywords:** hybrid manager, health systems, management training, tertiary hospital, Western Cape, low- and middle-income countries

## Abstract

**Background:**

Effective leadership and management are required for quality health services in low- and middle-income countries (LMICs). As in higher income countries (HICs), clinicians in LMICs often transition from a purely clinical to a hybrid role with managerial responsibilities, without management training. These clinicians with dual responsibilities are referred to as hybrid managers (HMs). This study explored opportunities and challenges experienced by HMs in a tertiary academic hospital in South Africa.

**Methods:**

A qualitative study was conducted using purposive sampling. In-depth interviews were conducted with 12 clinicians who were in a managerial role for over 12 months with no formal management training.

**Results:**

Hybrid managers experienced minimal support when first stepping into the job and articulated the large administrative burden. In an academic hospital, there is the double burden of bureaucracy from the hospital and the university, with the expectation to conduct research, teaching and training. However, direct patient interactions and clinical service also brought opportunities. This included knowledge of the business needs from a clinician’s perspective, as well as meaning-making, de-stressing and job satisfaction.

**Conclusion:**

Hybrid managers in this LMIC setting share challenges experienced by HMs in HICs with respect to identity, competing work priorities and the complexity of research and teaching responsibilities.

**Contribution:**

Hybrid managers in tertiary hospitals require support with adequate orientation towards the administrative processes, budgeting, human resources management and workflows in the hospital and university. Mentorship is key, as is training towards a mind shift change for HMs to truly embrace the role.

## Introduction

The World Health Organization (WHO) has identified the importance of strengthening health systems to improve health outcomes in developing countries.^1^ Complex health systems need effective management.^2,3^ Clinicians in low- and middle-income countries (LMICs) especially need to have strong management skills;^4,5^ however, they often find themselves managing services without the benefit of specific training for this role.^6^ These hybrid managers (HMs), who perform both clinical and managerial roles, have often had to ‘learn management on the fly’,^7^ as management is not a skill set that forms part of routine clinical training. The concept of an HM in healthcare is that of an individual who practices clinical medicine and also engages in the management of services.^8^

In the developing world, most health managers are clinicians in the services who then also start performing management tasks, without having received additional training in the sphere of management.^9^ In spaces where training is provided, it is usually centred on the tangible competencies and does not usually focus on the more intangible competencies required, such as social awareness and emotional intelligence.^6^ Literature from the district health system in the Western Cape (WC) shows that there has been a paucity of upskilling in the realm of managing people.^10^ There is very little research on this topic in a hospital setting.

Low- and middle-income countries commonly adopt formal management and leadership training programmes in the health setting.^6^ This is more suited to formally appointed health service managers and is usually more focused on the cognitive aspects. Other types of training are on-the-job training and action learning.^6^ An example of workplace-based learning combined with a formal training programme in a LMIC is Oliver Tambo Fellowship Programme at the University of Cape Town (UCT). This has been developed specifically for healthcare managers who have to manage services in a complex health environment.^11^

According to Mintzberg, ‘management has to be practiced as a craft, rooted in experience, and an art, dependent on insights’.^12^ Managers have various responsibilities including the management of clinical services, financial and human resource management, as well as navigating relationships with various external role players.^6^ Leadership and management are both important; however, they are different skill sets.^13^ Leadership is conceptualised as a dynamic process through which individuals are empowered to perform effectively within a particular contextual framework.^6^ Transitioning from a full-time clinician to an HM not only has many challenges but also presents opportunities for the HM.^8^

This study aimed to investigate the management experiences of clinical staff in a tertiary academic setting, who, after serving exclusively in clinical roles, had transitioned into managerial positions for a period exceeding 12 months. Effective management requires managers to be equipped through the integration of three forms of intelligence: cognitive, emotional and social.^6^ This study aimed to explore how well-equipped HMs are to manage people.^14^ Most HMs in this type of setting do not have specific formal training in management, and the study sought to focus exclusively on this group.

## Research methods and design

### Study design

This was an exploratory study done following a qualitative research approach taking into account the contextual realities of the HMs and trying to understand how clinical staff experience their managerial role.

### Setting

The research was performed at Groote Schuur Hospital (GSH), a tertiary academic hospital in the WC, South Africa (SA). This hospital provides patient care across most clinical disciplines – for acute and chronic conditions. It receives referrals of high acuity patients, from clinics, district and regional hospitals.

While senior managers have a full-time management role, middle managers in this context are typically HMs who maintain significant clinical responsibilities. They generally oversee teams ranging from 5 to 50 personnel, and are accountable to senior leadership. Senior clinicians hold joint appointments, with dual accountability to the WC Department of Health and Wellness through the hospital and to the UCT. Both the hospital and the university function as complex institutions, having a wide span of functions and responsibilities. They are each governed by different administrative systems. Hybrid managers within this setting are tasked with service management, as well as engaging in research, teaching and training activities.

### Study population and sampling strategy

The study population comprised HMs at GSH who perform a managerial role alongside their direct clinical roles across the spectrum of clinical disciplines. Purposive sampling with snowballing was used to select 12 participants across different clinical departments, age groups and with a range of years of management experience. Snowballing refers to a sampling methodology whereby study participants assist in recruiting other study participants from their own networks. Inclusion criteria were clinical staff who were in a managerial position for over 12 months and who had studied medicine or were in an allied health discipline. Clinicians who had a formal diploma or higher-level training in management or who trained in Psychology, Psychiatry or the Social Work discipline were excluded, as these disciplines receive elements of emotional and social intelligence during their training. Full-time managers or those clinicians who had less than 12 months managerial experience were also excluded.

### Data collection

The researcher conducted in-depth interviews (IDIs) in English. The researcher was a non-clinical Medical Manager at another academic tertiary hospital in the WC. A discussion was held prior to the interview to inform participants that this was an independent research study not commissioned by the hospital. Furthermore, this research was not part of normal working arrangements, and any information obtained during the interviews would not impact on the performance management of the staff member – either negatively or positively. The participant information sheet also reinforced this. Researcher reflexivity was cultivated by the researcher documenting her own beliefs regarding the research topic, to elucidate and manage any bias that may impact on the findings. These were discussed with the researcher’s supervisor during the time of data collection, analysis and write up.

During the IDIs, an interview guide was used to assist the interviewer. Data collection was done in a confidential space and at a suitable time when they were not expected to work. To ensure credibility of the research, the researcher documented verbal and non-verbal data. All participants provided written consent for the audio recording of the interviews. A digital recorder was used to audiotape the interviews and a mobile phone application for a backup recording. Brief notes were taken. It was hoped that rich data would emerge about the insights of HMs into their management role.

The researcher began data collection in February 2020 with face-to-face interviews. Eight interviews were completed by March 2020. Each interview was 40–65 min. Thereafter the researcher could not continue with the research as the coronavirus disease 2019 (COVID-19) pandemic spread to SA and the WC. The researcher was then assigned to overseeing the COVID-19 preparedness plan and services in her hospital until the middle of September 2020. Other researchers also faced similar limitations during the pandemic.^15^

The researcher restarted the interviews in October 2020. Four interviews were concluded in this month – one was in person, one was online on Microsoft Teams and two were telephonic. Written consent was obtained prior to the interviews.

The researcher used the following exploratory questions in the interview guide:

- Please share with me how your career-pathing led you to be here now?
- Please tell me about any training that you have had with regards to your managerial role:
  ■ In particular, what training have you had with regards to managing people?
- What is your experience of managing people while in your current role?
- What aspects of managing people do you enjoy and what do you find most challenging?
  ■ Let us start with what you enjoy
  ■ What do you find challenging?
- In what ways do you think that your clinical background and work gives you an advantage when you are performing your managerial role?

For the last four interviews done during the pandemic, the researcher also asked whether the participants had to adapt any of their usual practices during the pandemic. This was an important element to bring in, as many clinicians had to work differently during the pandemic.

### Data analysis

A transcriptionist transcribed the data verbatim after each interview. The researcher initially commenced a manual process of identifying themes. However, this process was found to be laborious, thus NVivo 12 quality data analysis software (QSR International, Melbourne, 2018) was utilised to increase efficiency and to enhance the rigour and validity of the study through systematic organisation of the data. The researcher read the transcripts and listened to the interview recordings more than once to surface new aspects.

Thematic analysis was done. Peer feedback from the supervisor was received on the first three transcripts that concurred with the initial thematic nodes that were drawn out. However, after the COVID-19 period of research absence, the researcher identified additional thematic nodes, and in some areas, multiple themes could be collapsed into one. The researcher used the NVivo 12 mind map modality to organise her thoughts about the emerging themes.

The analysis became easier with each subsequent script, and new insights reduced from the 9th script. The thematic analysis of the earlier transcripts was revisited after the break imposed by the COVID-19 lockdown.

### Ethical considerations

The researcher followed the ethics principles outlined in the University of the Western Cape (UWC) Policy on Research Ethics, and approval was obtained from GSH. Ethics approval was obtained from UWC Faculty of Community and Health Sciences Higher Degrees Committee, UWC Biomedical Ethics Committee (BM19/9/11) and UCT Human Research Ethics Committee (883/2019). Written informed consent was obtained from all the participants.

## Results

The study sample consisted of 12 HMs – 7 females and 5 males with equal numbers of doctors and allied health professionals (Table 1). Most were in the age group 40–49 years. Participants had some experience (the inclusion criteria required at least 12 months of experience), with the majority having above 5 years of managerial experience.

**TABLE 1:** Characteristics of participants.

Quotation reference in article	Age category (years)	Gender	Management experience (years)
HM1	40–50	F	< 5
HM2	40–50	F	5–10
HM3	50–60	M	10–15
HM4	50–60	M	> 20
HM5	60–70	M	> 20
HM6	40–50	F	15–20
HM7	40–50	F	< 5
HM8	40–50	F	10–15
HM9	50–60	F	15–20
HM10	40–50	F	15–20
HM11	40–50	M	15–20
HM12	40–50	M	5–10

HM, hybrid manager; M, male; F, female.

**FIGURE 1:**
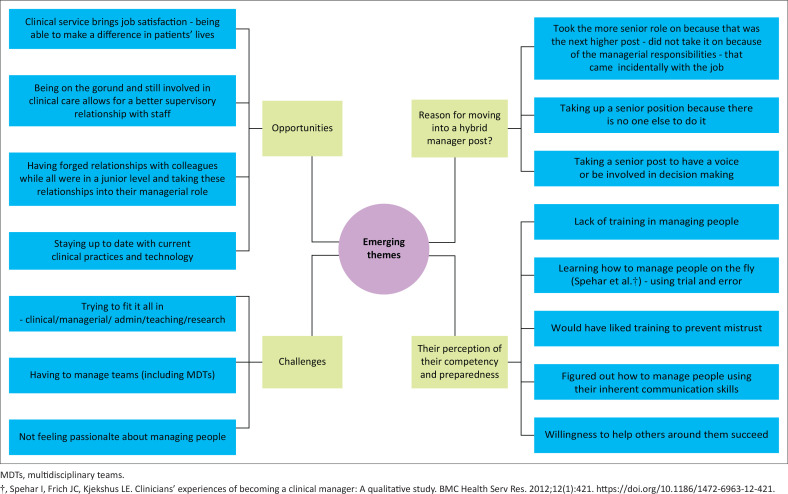
Mind map of initial thematic analysis.

There were many themes that emerged during the initial analysis. See Figure 1. These were then organised and grouped into themes and sub-themes See Table 2.

**TABLE 2:** Areas of enquiry and themes emerging from data.

Themes	Sub-themes
1. Reasons for moving into a hybrid manager role	1.1. The next higher role came with managerial responsibilities 1.2. There was no one else to do it 1.3. To have a voice and be involved in decision-making
2. Hybrid managers’ perceptions of their competency and preparedness	2.1. Learning how to manage through trial and error 2.2. Applying their inherent interpersonal skills in management 2.3. The power of mentorship and support 2.4. Would have liked to have training
3. Opportunities of being a hybrid manager	3.1. Clinical service brings job satisfaction 3.2. Being on the ground assists with supervisory relationship with reporting staff 3.3. Using existing relationships built up over time 3.4. Staying up to date with clinical practices and technology 3.5. Supporting other clinicians
4. Challenges faced by hybrid managers	4.1. Balancing clinical versus other demands of the role 4.2. Managing people and multidisciplinary teams 4.3. Negotiating the bureaucracy 4.4. Lack of support

### Theme 1: Reasons for moving into hybrid manager role

#### The next higher role came with managerial responsibilities

Many HMs did not choose to be managers specifically, but found that, as they progressed through their career, higher positions tended to involve management. Most participants were accepting of this; however, four participants shared that they felt a bit resistant to this at the outset:

‘I applied for the … [*senior*] post, and I was successful in that interview. Initially I didn’t want to, because I didn’t want to take on the responsibility of managerial. I still wanted to be a clinician, and it was so difficult to make the decision of how do I do this, because I know I’m going to lose patient care. It almost stopped me at one point of actually going through the interview itself, but I said there must because a reason for it. So I think that kind of – being able to really still be a clinician, and not almost take on too much, but as a manager you’re going to.’ (HM7, F, < 5 years managerial experience)

A few participants expressed their feelings of inadequacy for this more senior role initially but were concerned that another such opportunity would not arise for them:

‘And in fact, opportunities will only be created if somebody leaves their post.’ (HM6, F, 15–20 years managerial experience)

#### There was no one else to do it

Some had no aspiration to progress but felt obliged to put their hand up when the leader of their area retired. Some academic departments appoint a head on a rotational basis for a specified number of years – one HM felt pressured into accepting this job in spite of their existing challenging clinical load:

‘I didn’t particularly aspire to being the head …. So, being the head … wasn’t the thing. It was … having a head … for this department. So, I don’t feel strongly about being that person, and I was very happy to move over if there was someone else who did fulfil those criteria, but there wasn’t and there isn’t … I just wanted this department to be functional. And it’s difficult to be a functional department when you don’t have a head.’ (HM9, F, 15–20 years managerial experience)

#### To have a voice and be involved in decision-making

Two HMs became managers to participate and assist in the planning, delivering and improvement of clinical services:

‘I think the one thing that always pushed me, I’m passionate about patients, but obviously as you go along, you realise decisions are made at higher levels, and as long as you’re on the ground, you don’t get to give all the input that you would like.’ (HM2, F, 5–10 years managerial experience)

### Theme 2: Hybrid managers’ perceptions of their competency and preparedness

Many of the HMs shared that they felt inadequate when they entered their initial managerial space. A few expressed that they would still like to receive management training and orientation to administrative tasks.

#### Learning how to manage through trial and error

None of the participants had acquired formal management qualifications. Several had attended shorter informal courses organised by the hospital. Most of these were one- or two-day courses covering conflict, change and project management and also included technical training such as information technology (IT) skills. A participant shared how they focused on self-learning after hours, trying to upskill themselves in the area of leading and managing. Most of the participants indicated that their learnings were based on trial and error:

‘It changes constantly. So one week you’re fine, one month you’re fine, then for the next six months you feel like you’re in a war zone. So I think those are things that I find very challenging, that if it’s not related to my job per se, it’s other things. So just managing people and understanding people, that I have found extremely challenging. I think it comes a little bit in your training, but ja, you need a whole other degree for that.’ (HM2, F, 5–10 years managerial experience)

Role modelling was highlighted as a valuable learning method. Several participants shared that they learned by watching the people management skills of colleagues. However, one participant indicated that they could either look to their leader’s behaviour as something to aspire to, or in some cases, something to distance themselves from:

‘I have multiple other leaders around me who I have gleaned things from, both positively and negatively.’ (HM12, M, 5–10 years managerial experience)

#### Applying their inherent interpersonal skills in management

Hybrid managers were asked to think about their inherent interpersonal skills and to reflect on whether these aided them in their managerial role. Many shared that certain innate characteristics such as courage, persistence, self-confidence and resilience enabled them to perform their managerial role more readily:

‘[*T*]here were many times when I thought oh, this is not for me, it’s not going to work out, and if I wasn’t persistent then it wouldn’t have. So persistence I think is very important, hard work obviously and resilience. This place can wear you down in many ways. … because you are doing the clinical service and you’re dealing with a lot of bureaucracy there, and the academic side. But I think also courage is important, taking risks, innovating, finding entrepreneurial ways of doing things.’ (HM3, M, 10–15 years managerial experience)

#### The power of mentorship and support

Many of the participants were grateful for the support they receive from others in their space. Although at times this came from their line manager, more frequently support was received from other colleagues, peers or mentors outside their clinical areas. Support could be in the form of someone who had an open-door policy where thoughts could be ‘bounced off’ the other to work through a scenario before tackling it. Hybrid managers engaged with various mentors depending on what type of challenge they were facing. Several participants felt supported if their direct line manager showed an interest in their personal life. Three HMs shared appreciation for a specific mentor whom they felt actively looked for opportunities for them to advance their careers:

‘So when I came here, it was … [*X*] who really was the reason I came here, and when he heard that I was looking to come back …, he got in touch with me actually via some people he knew … And he called me one day out of the blue … And all credit to … [*X*] as well for driving the whole thing … he was so passionate about the whole thing, … and so things stay with you. And so you know, we try and do the same now … I’m still in exactly the same situation now with very superbly bright, young people who have just got their specialist exams.’ (HM3, M, 10–15 years managerial experience)

#### Would have liked to have training

Several participants indicated that they were currently faring well with the managerial role. However, training and orientation into the management space would have been welcomed in the beginning:

‘It would have made the world of difference, and it would have eliminated a lot of embarrassing mistrusts. I mean, part of what I do now is budgeting, you know, ordering of resources, but I was never really taught to do that. It was just something that I had to learn over the years, by trial and error … Or even if it was something informal, where someone sits you down and says hey, this is what’s going to happen, it would have made a bigger impact.’ (HM1, F, < 5 years managerial experience)

A few expressed that they had created their own learning opportunities, often after hours, to support themselves. One participant often turned to their facility’s staff wellness programme to obtain advice:

‘And then what I have done is I have personally gone on managerial courses and things, just to assist me with that, and I found … there was ICAS, [*employee wellness programme*] and I just took it upon myself. They already know me, I phone them often because they can give me good input for the managerial part of it to assist, because a lot of the decisions that you’re making, it’s impacting on people’s lives.’ (HM2, F, 5–10 years managerial experience)

One participant talked about the specific skill set that is required to manage a team well, with all its complexity. They reflected that this type of training when one enters a managerial role would be beneficial to upskill HMs in the clinical space:

‘And so I think, because we’re such a relational occupation, that it would be very helpful of how to manage teams and at various points. Maybe not at medical school, but certainly I think in internship community service, as you start to be leading, how do you manage a team of fourth years, how do you manage a team of interns, how do you manage a team of registrars? That basic person management, people management, would be very helpful, conflict resolution type stuff, even just communication skills. Um, because that’s what we do all the time.’ (HM12, M, 5–10 years managerial experience)

Time pressures were cited as often affecting attendance at courses because of clinical and other competing demands. However, one participant indicated that they did not attend the available managerial training as they were more passionate about research than their managerial responsibilities:

‘I’m not really … passionate about administration and managing people. I’m not passionate about that. I do it because I have to do it.’ (HM3, M, 10–15 years managerial experience)

### Theme 3: Opportunities of being a hybrid manager

#### Continued clinical service contributed to job satisfaction for hybrid managers

Most participants reported that their main source of job satisfaction still came from their direct clinical involvement. They appreciated the chance to improve their patients’ lives; this was reflected as a very rewarding part of their job:

‘[*I*]t’s sometimes difficult. Because obviously you know, not everyone makes it, and not everyone – having to deal with family is difficult as well. So you try not to be too much, you know, like a one to one, but you still get to know if the brother or the mother or the father, whatever is coming in daily for a couple of days or a couple of weeks or whatever. You know, it’s difficult, but then also it is rewarding as well when they get from ICU, they go to the ward and they come back again in a couple of months’ time and say hey, I’m back.’ (HM11, M, 15–20 years managerial experience)

Several participants shared that they frequently felt pressured to stop doing clinical work because of the escalating volume of management tasks. They tried to resist this to keep doing most of their patient activities:

‘I think seeing patients keeps it real. It helps to remind you why you do what you do. So I like having some aspect of patient contact, which I do. It’s frustrating sometimes because that is often the first thing that goes, because there is someone else that can do that, whereas there is no one else that can do some of the other jobs … But I do try to keep clinical because I think that’s what keeps me grounded and it really also helps me to see what’s going on …’ (HM9, F, 15–20 years managerial experience)

One participant commented on their understanding of the importance of some administrative tasks, in that they did ultimately contribute to the overall wellbeing of their patients:

‘So you know, with all this paperwork, it can get very frustrating and get very annoying. I mean, there are days when I don’t see the sun because I’m stuck in an office all day, or stuck in meetings all day. But at the end of the day when we see how that makes a difference to the patient, it makes it worth it … Whether we’re ordering the toilet paper, … or whatever it is, we’re here for the patient, to make the patient’s lives a bit better.’ (HM1, F, < 5 years managerial experience)

Another participant shared how they would at times rather engage in direct patient activities as a way to destress, especially when the managerial role was taking a toll:

‘[*T*]hen I find, like my joy is my clinical work, then I just have a breather for a few days, and then I’m like okay, now I’m ready to tackle things again. I see what’s on the floor, I remind myself why I took this job. It’s to make the difference on the floor, so I’m like okay, then I go back in again.’ (HM2, F, 5–10 years managerial experience)

#### Managing from the floor and not only top-down

The majority of the participants felt that their involvement in the clinical services with their colleagues assisted them in their supervisory role as they were able to support their staff:

‘So I have always felt that for you to lead, you need to know what’s happening on the ground. So, a lot of the time when I go down onto the floor and I go and do the clinical work, I see what’s happening, and it makes it easier for me to understand their challenges.’ (HM1, F, < 5 years managerial experience)

Some participants shared that they thrived in engaging in direct patient care. It also allowed them to remain updated with current clinical practices and the advancement in medical technology. This meant they remained up to date with clinical practices and technology and were better informed in making relevant resource decisions:

‘If you became totally managerial and hands-off, and not involved in clinical, you’re not up to date with things. You lose your skill in it as well … But there’s new equipment, and there’s new … machines … So you need to keep your hands on all the different clinical side of things, as well as teaching, new consumables that come out, things like that.’ (HM7, F, < 5 years managerial experience)

All participants reflected that it was important to support others in their team. This could be directly to their subordinates, other clinical staff categories or external students. As a renowned academic institution, the hospital drew numerous external students – both undergraduate and postgraduate – from outside the province and even from other countries beyond SA. The participants shared how they implemented support mechanisms, tailored to direct observation of the need. One participant preferred to abandon the usual system of hierarchy and focused instead on providing emotional support to junior staff when it was appropriate:

‘[*S*]o speaking to some other managers, they would say keep away, or don’t get too involved, or you know, just – but I’m not that type. So if I can do something, then I will do it. So especially with the registrars rotating, I would give particular attention to those that … were living alone and away from home … I would have coffee with them, I would sit and have lunch with them, you know. They just seem to need a bit more support than the others.’ (HM10, F, 15–20 years managerial experience)

#### Supporting other clinicians

Exposure to managerial responsibilities was mentioned as a valuable method to support and upskill subordinates, via the attendance at managerial engagements or by delegating specific tasks for a period of time. A different manager spoke of providing support by ensuring that their subordinates took their time off to recuperate after hours. Academic support and career progression advice was also needed. The ad hominem rank promotion process is tricky to navigate. There are several criteria to fulfil, for example, national and international standing, number of publications and community engagement. This often takes a significant number of years to attain. Clinical staff are often not clear on what the full list of criteria is, prior to applying:

‘To help the young registrars and consultants … when I started, the first thing I did was I emailed the [*ad hominem*] promotion form to everybody and I said start filling it out. Start thinking about what you want to do, but even just for your performance assessments, put it in this format. So by the time that – when it comes time to update your CV, you’re not moving in darkness.’ (HM10, F, 15–20 years managerial experience)

A few participants tried to create a safe space for junior staff to share about any difficulties they encountered in the workplace regarding clinical service, academic pressures or even interpersonal matters:

‘[*S*]everal people have commented that they’ve really appreciated me being around, just because they can bounce stuff off without feeling they’re going to have their heads bitten off. And I have taken a far greater interest in the senior registrars in terms of their training and who they are, both in what they’re supposed to do in terms of their medical training but also them personally.’ (HM12, M, 5–10 years managerial experience)

### Theme 4: Challenges faced by hybrid managers

#### Balancing clinical versus other demands of the role

All HMs indicated that they struggled to complete the different tasks of their work. Besides a substantial clinical workload, they had to do administration, manage people, teach, train and research:

‘So that is very challenging when you have to do – you have other meetings to attend to, managerial things that you have to do. But then there’s just this absolute amount of patients that you need to get to … So initially when I started, I found it extremely challenging to juggle being a clinician and being a manager.’ (HM2, F, 5–10 years managerial experience)

Some participants successfully delegated tasks; however, many could not find a suitable person to delegate tasks to:

‘I’ve also learnt that there are some things that I just can’t do, and that I have to rely on people around me to do. So I think I’ve become better at delegating, and initially I was quite unsure of myself, especially when I wasn’t appointed [*managing the department in an acting role*], and I was very unsure of myself. Now, I’m more sure of myself and more confident about my position. It’s kind of easier for me to delegate and ask other people to do things and see what needs me and what can be done by other people.’ (HM9, F, 15–20 years managerial experience)

Half the HMs did not manage to complete all their job tasks during the allocated work schedule and often had to work in the evenings or on weekends:

‘I do a lot of work over weekends and at night and I work till 11 every night probably, so that’s what I do at home. But that’s also international work and writing as well. That is difficult.’ (HM5, M, > 20 yrs managerial experience)

Despite being an academic hospital, not all HMs aspired to perform research. One participant indicated that their preference was to do the clinical work; however, their peers also expected them to publish:

‘So in our department, there is a big drive towards publications. We are a machine when it comes to publication and putting ourselves on the map. I am quite willing to not do it, but I’m willing to do your work for you, but that doesn’t count.’ (HM8, F, 10–15 years managerial experience)

Most participants expressed their desire to perform all their different work components; however, one of the HMs reflected their sense that the overall workload expected from them was too much:

‘I try to have a better work life balance, but I struggle. I think that what is expected in terms of load is not really reasonable.’ (HM9, F, 15–20 years managerial experience)

#### Managing people and multidisciplinary teams

All participants shared that they needed to supervise staff and, in addition, some also had to manage a multidisciplinary team, which needed a specific skill set. These teams were often not only clinicians but also non-clinical and support staff:

‘I have to take that plunge and responsibility. Because it’s not only us, we are in a department. So there’s nursing staff, there’s cleaning staff, there’s porters, there’s doctors, so you have to interact. So it’s internal management, but also managing everything around the clinic and it’s not always easy.’ (HM4, M, > 20 years managerial experience)

Several participants did not like to handle interpersonal conflict but acknowledged their responsibility to do so when required:

‘One of the biggest challenges for me I think is taking things too personally and the issue of conflict in terms of dealing with difficult staff.’ (HM12, M, 5–10 years managerial experience)

A particular participant described how they would encourage their staff to follow a new process that had been introduced by the hospital. This HM would inform their team that these instructions arose from higher up in management to avoid conflict with their team:

‘I’ll say look, I’ve been instructed by people above me, you know. And it’s not negotiable.’ (HM11, M, 15–20 years managerial experience)

Two participants expressed that they were passionate about patient care and not particularly about the management of staff. Another indicated that some heads of departments may be extremely successful in academia; however, they did not have the appropriate management skills to run a department made of people:

‘But I mean, you see it, I think, in all these big hospital institutions, that there are guys that are very, very high up academically who’re just the most horrendous managers because they’re brilliant academically, but just not people-managing people.’ (HM12, M, 5–10 years managerial experience)

#### Negotiating the bureaucracy

All the participants indicated that their administrative duties were additional and secondary to their primary clinician role. They shared their frustration at the cumbersome processes they had to follow, which were felt to be unnecessary bureaucracy and extremely time consuming. Several HMs reported the need to acquaint themselves with the administrative processes, including procedures for ordering consumables and arranging equipment maintenance:

‘No, we don’t have major support … if I can give an example, we had a laptop that was stolen. It belongs to our … machine. It has taken almost nine months for them to replace that stolen item, because again, we don’t understand always the processes. Like nothing had ever gone stolen before. No one had explained it to us, and often I was then moved round to about four different departments, from security, down to the actual hospital equipment group.’ (HM6, F, 15–20 years managerial experience)

Several participants indicated that they were frustrated by the volume of paperwork that was expected from them for the human resources department and also shared their challenges in finding out what the correct process was to be followed. One participant felt that the various forms that they had to complete did not assist them in managing their team, but took an unreasonable amount of time that could have been used to do other important tasks:

‘So increasingly there is so much documentation that has to be completed for everything, that finding your way through that is really – it’s difficult, and sometimes I just need a sensible person with some judgement. You know, and sometimes that’s hard to find because everybody is so busy filling in their forms.’ (HM9, F, 15–20 years managerial experience)

#### Lack of support

All participants reflected on periods when they felt unsupported at work. For several, this occurred at the time of appointment. There was often no transition period, especially if the job had been unfilled for a while, before they started. It was suggested that a handover period would assist to orientate new HMs to their managerial role, as well as allow for the explanation of various processes that needed to be followed:

‘So it would have been nice if in fact a transition phase, because often when you find you move up to another position, that person who occupied that position had left already. There is no supervisor to whom I can actually report to, could show me this is what is required. So I’d have liked maybe a better sort of transition. For at least a month, someone could have at least said well, these are the forms that you needed to fill in for leave, these are the forms that you needed to fill in for SPMS, these are the forms, you know for your budgets, for your overtime. But there were no systems that were there in place.’ (HM6, F, 15–20 years managerial experience)

For others, the lack of support was ongoing. One participant suggested that the lack of support from their direct supervisor could have been because of them being inundated with various tasks. Another felt that, as senior staff, they were overlooked for support as the need was not recognised:

‘I think it’s just, most people require support. They just actually need encouragement. As simple I think as humans are, if someone just actually thanks you for doing your job, and for doing it well, reasonably well, not just brilliantly, that people would be far more happy, satisfied, capable, but actually that someone noticed what I did, when I did something that I was supposed to do, rather than something extraordinary.’ (HM12, M, 5–10 years managerial experience)

## Discussion

This research study set out to explore the experiences and insights of clinicians who had transitioned from a more technical clinical role to that of an HM role, where they are expected to perform managerial functions besides their clinical responsibilities. There are only a few studies on HMs in LMICs – these all describe primary care services in the context of a district health system. There are some similarities and differences between the experiences of HMs in a tertiary academic hospital and those in the primary care system.^13,16^

### The path to becoming a manager in a tertiary hospital

In this study, set in a tertiary academic hospital, none of the participants stepped into the role because they were drawn by the management aspects. The management responsibilities were seen as an incidental addition to their main promotion role to senior clinical staff member. This is in line with studies in other healthcare environments where different pathways into the managerial arena are described.

The managers in this study would be considered to fall into the category of ‘incidental’ HMs as opposed to ‘willing’ HMs.^17^ The consequence is that they are slow to embrace the managerial identity, which puts them at odds with their clinical identity.^18^ As shown in this study, the two roles can compete for time rather than be complementary. According to Witman et al.,^19^ HMs find it difficult to remain credible in the two different arenas – that of medicine and that of management – this further dilemma resonates with the findings of this research. Spehar et al.^20^ found that in spaces where HMs felt obliged to take up the role, this affected the manner in which they tackled the management duties, as their patient-facing work was felt to be more valuable than their management tasks. If managers are ambivalent and do not fully embrace their managerial responsibilities, as described in this study, there will be an impact on the services they manage.^21^

The greater question for health systems is whether this approach to career-pathing is appropriate or if other pathways should be forged. The common practice of career progression to an HM role should perhaps be adapted, as not all clinicians and researchers make for great managers. This raises the importance of presenting management as a deliberate career path for clinicians, rather than an option for clinical promotion. A different approach would be to provide spaces where clinicians who are not yet managers could be orientated to management practices. Those who exhibit interest and show evidence of early management skills could then be upskilled with specific training and mentorship. It would also be important for leadership development in an institution to be planned taking into account its contextual environment.^22^

### Training for hybrid managers

This study purposefully excluded HMs who had formal management training, as it was interested in the experience of most HMs in this setting who, as with HMs in other countries, do not have formal management training.^23,24,25^ Like many HMs reported in the literature, in this setting, and without formal training, HMs felt ill prepared and had to learn management ‘on the fly’.^7^ They shared that they would have preferred having some managerial training before taking on the role. A few of the HMs had obtained informal training after appointment, and some were fortunate to have benefited from mentoring.

In this study, most participants had more than 5 years managerial experience, with half of the participants having been in a managerial role for more than 15 years. For such a group of HMs with a fair amount of experience, training should be designed to enhance their existing technical and management skills.

Hybrid managers require upskilling, not only in technical aspects but crucially to support their ability to adapt and function in a complex health system.^11^ Training towards a mind shift change is needed for HMs to truly embrace the managerial role as a primary function of their job. According to Mintzberg,^12^ an effective manager requires three essential elements – art, craft and science:

‘Art brings in the ideas and the integration; craft makes the connections, building on tangible experiences; and science provides the order, through systematic analysis of knowledge.’ (n.p.)

It would be important for senior health managers to ensure that HMs are adequately equipped in these different aspects of managing.

### Support for hybrid managers

This research study highlighted that newly appointed HMs often experience inadequate support, without effective orientation. Commonly, the post was already vacant for a while before the new HM was appointed. Any manager, whether experienced or not, would require an adjustment period as they take on a new role.^12^ This would also apply for clinicians in a hospital.^26^ Senior staff should not assume that clinical staff will be able to find their own way in a new space. Instead, it would be important to provide adequate support for clinical staff as they transition to a leadership role. The report of the *UCT Enquiry into the Circumstances Surrounding Professor Bongani Mayosi’s Tenure* (p. 126)^27^ strongly reinforces this sentiment:

‘It was also emphasised that this ‘sink-or-swim’ approach has sometimes been explained as being based on the belief that university insiders do not need induction, as in the case when a staff member moves from one department to another. This short-sighted belief needs to be reviewed, given the many personal and professional variables at play in any such transition.’

Despite the lack of managerial training, many HMs in this setting were able to adapt and manage the various tasks within their role. A few indicated that being inherently resilient is a personality trait that assists them to perform better. Building up their personal resilience has been recommended as a component of HM development.^28^ The ability to cope with the job may also be supported by the significant amount of workplace experience that these HM bring to the job in this setting. The path to being a senior clinician in a tertiary setting is long, and advanced clinical training is embedded in the workplace. The implication of this is that they have a wealth of experience to draw on in reflective workplace learning.

It was interesting to note that many of the HMs who did not themselves receive adequate support went out of their way to set up support mechanisms for their juniors. Training alone is not sufficient to equip HMs; it must be strengthened with the implementation of adequate support structures which are flexible and can assist during challenging periods.^6^. An example of a crucial element of support is mentoring and should include safe spaces where HMs feel able to reach out to more experienced managers to ‘bounce off’ ideas, without worrying about being ridiculed.^28^

### Opportunities inherent in the hybrid role

The hybrid model, if adequately supported, offers inherent opportunities to address some of the current weakness in health management in LMICs.^26^ High staff turnover, including that of managers, has been linked to low job satisfaction. As discussed next, clinical work can boost job satisfaction. Poor performance has been linked to low staff morale fuelled by a sense that managers are disengaged from the reality of service delivery on the ground. Hybrid managers, still involved in service delivery, are in a position to demonstrate their understanding of the situation, and this gives context to their managerial action.^29,30^

### Value of clinical work in job satisfaction

Hybrid managers highly value their clinical role. All participants expressed that their job satisfaction and sense of fulfilment primarily stemmed from their patient-facing responsibilities, rather than from administrative or managerial tasks. According to Witman et al.,^19^ the job satisfaction of HMs decreases as they perform less direct patient care. The HMs in this study exhibited a similar tendency, reporting that they did not derive job satisfaction from managerial or administrative duties. Moreover, participants noted that engaging in clinical work often served as a source of stress relief.

### Value of clinical work in supervision and management

Hybrid managers indicated that engaging in patient-facing work enabled them to maintain a presence on the clinical floor. This helped them to understand the workspace of their subordinates and improved the quality of the supervision. It also gave them an opportunity to keep in tune to evolving clinical practices and technology. Hybrid managers can have an advantage over full-time managers in having more support from other clinicians, by virtue of their position and their perceived competency.^8^ One opportunity that emerged as important in this study is the perceived respect that HMs felt from their subordinates based on their clinical expertise and research portfolios.

### Importance of context and the complex tertiary setting

The understanding of context is an essential element in developing leadership in healthcare.^24^ Compared to their previous focus on purely clinical duties, clinicians have taken on an expanded scope of responsibilities, including management. In addition, the ‘manager’ role in healthcare has become associated with bureaucracy.^18^ Hybrid managers will respond differently to these demands, depending on their environment.^31^ This study showed that, while some experiences of HMs at a tertiary level hospital were similar to those described in the literature, in other settings, there are important differences.^2,32^ Coordinating the three domains of research, teaching and clinical service introduces an added dimension of complexity. This specific challenge is largely absent from existing literature on HMs in LMICs, which primarily focuses on district health services. In contrast, hospital settings present a distinct and multifaceted form of complexity.^10,33^ A tertiary academic hospital has even more stakeholders working in one geographical location and who are connected in various ways. In a large organisation, there are many more departments, each lead by its own head, with whom the HM needs to engage.

This study highlights a challenge that, to the researcher’s knowledge, has not yet been documented in the literature concerning LMICs – the double burden of bureaucracy that HMs have to manage in an academic hospital – that of the academic institution of a university and the service delivery institution of the Department of Health. The new HM often has to make their way through many difficult and time-consuming administrative processes in two separate organisations without any clear explanation or orientation. These parallel processes are in place, for financial, human resource and other spheres, and the HM is expected to ensure that governance is upheld in both realms.

All participants articulated the large administrative burden, somewhat accentuated by a lack of orientation to the administrative process flows within the hospital. These HMs attempted to fulfil these commitments. However, several participants reflected that so many of the tasks did not add value. Hybrid managers perceived that some of these unnecessary bureaucratic processes should be buffered by the non-clinical institutional managers, instead of just being handed down the line to HMs who are trying to balance clinical and managerial tasks. The wellbeing of clinical staff would be enhanced by the provision of appropriate administrative support.^34^

### Strengths and limitations

This qualitative study did not aim for generalisability, but rather sought to elicit meaningful insights. Although the sample size was small, it still demonstrates some interesting reflections about the experiences of HMs in a tertiary hospital which may apply to other institutions in the WC tertiary academic complex, as well as in other provinces, for example, Gauteng and KwaZulu-Natal. Something that can be seen as a limitation is the fact that eight interviews took place in the first 2 months of 2020, before the COVID-19 pandemic reached SA. Thereafter, the researcher stopped data collection as the first wave commenced in March 2020 and restarted in October 2020. It is acknowledged that the experiences shared by the final four participants may have differed had the interviews been conducted prior to the onset of the COVID-19 pandemic.

### Recommendations

A distinction should be made between clinicians who recently became HMs and seasoned HMs who have been in this role for many years. Technical and managerial skills already acquired should be acknowledged with training tailored to their particular needs.

Hybrid managers require sufficient orientation about the administrative processes and workflows within both the university and hospital environments. This should include a wide array of concepts, for example, finances, managing people including labour relations processes, organisational values and the role of the hospital within the broader provincial and national settings. An understanding of the equipment planning and procurement processes in a highly specialised service is crucial. The facility could develop a training programme including all these elements to equip staff.

Support should also be provided in a proactive manner to assist the individual with career planning. Clinicians often struggle with understanding how to move through the ad hominem academic rank promotion process. This is especially relevant in SA universities where the professoriate is on the decline.^35^ Hybrid managers who wish to pursue academia would feel supported if they were provided with opportunities to move beyond the clinical into some managerial spaces in the hospital and university.

The more common scenario is for senior clinicians to become HMs as they are furthering their academic and clinical career. They do not usually do so because of a drive to step into a manager role. These HMs usually have no prior exposure to the managerial environment. One possible way to provide this opportunity through the creation of an enabling environment is to second interested clinicians to the management space for an allocated time to shadow a medical manager who is a full-time manager. Partnering clinicians with an appropriate mentor, with whom they feel at ease to bounce ideas off, could assist them in developing some managerial skills and start a reflective learning practice. This is currently being implemented at Red Cross War Memorial Children’s Hospital in the WC. This would provide the clinician with an on-the-job learning opportunity to begin developing the managerial skills needed when they step into an HM role further on in their career. This early exposure may assist with the development of the managerial identity.^36^ Training towards a mind shift change is needed for HMs to fully embrace the managerial role.

In this tertiary academic context, there are certain parallels with primary health care settings – most notably, the transition of clinicians from exclusively clinical roles into managerial positions without the benefit of additional training. However, this study showed that there are additional layers of complexity. These findings may be similar in other SA academic settings and beyond. Further research should be explored in this setting.

## Conclusion

None of the HMs in this study assumed their roles out of an interest in the managerial aspects of the position. As a result, they often struggled to fully adopt a managerial identity. The consequence thereof is that they do not fully embrace the manager identity. Hybrid managers face a large administrative burden. In addition, in an academic hospital setting, there is the somewhat double burden of bureaucracy, as clinicians have responsibilities to two complex organisations – the hospital and the university. Hybrid managers reported their difficulty in balancing the different demands of their job.

By selection design, none of the HMs in this study had received any formal management training before embarking on the role. This is the norm for HMs. Yet they felt unprepared and had to learn management through trial and error. They expressed that they would have benefited from management training, but despite this, many HMs seemed to cope with the demands of the different aspects of their job.

There are also particular opportunities in being an HM – job satisfaction was derived from their involvement in direct clinical work which brought a sense of fulfilment.
